# Lifestyle Adherence and Metabolic Control in Adults with Type 2 Diabetes: Development of a Lifestyle Compliance Index

**DOI:** 10.3390/diagnostics16183068

**Published:** 2026-09-21

**Authors:** Bianca-Lăcrimioara Petca, Paula-Alexandra Popovici, Andreea Diana Igna, Timea Claudia Ghitea, Mihaela Simona Popoviciu

**Affiliations:** 1 Doctoral School of Biological and Biomedical Sciences, University of Oradea, 1 University Street, 410087 Oradea, Romania; criste.biancalacrimioara@student.uoradea.ro (B.-L.P.); cipleu.paulaalexandra@student.uoradea.ro (P.-A.P.); igna.andreeadiana@student.uoradea.ro (A.D.I.); 2 Pharmacy Department, Faculty of Medicine and Pharmacy, University of Oradea, 1 University Street, 410087 Oradea, Romania; 3 Department of Preclinical Disciplines, Faculty of Medicine and Pharmacy, University of Oradea, 410028 Oradea, Romania; mihaela.popoviciu@didactic.uoradea.ro

**Keywords:** albuminuria, cardiovascular risk, chronic kidney disease, lifestyle adherence, Lifestyle Compliance Index, metabolic control, obesity, risk stratification, triglyceride–glucose index, type 2 diabetes mellitus

## Abstract

**Background:** Lifestyle modification is central to type 2 diabetes mellitus (T2DM) management, but adherence is difficult to assess in routine care. We developed a pragmatic Lifestyle Compliance Index (LCI) from routinely collected behavioral variables and examined its cross-sectional associations with cardio-renal-metabolic measures. **Objectives:** To describe the LCI, compare outcomes across adherence categories, evaluate adjusted associations, and quantify incremental discrimination for prevalent metabolic and renal abnormalities. **Methods:** Adults with T2DM attending a specialized outpatient diabetes clinic in Romania were assessed using a standardized clinical questionnaire and routine laboratory data. The LCI combined seven binary behaviors and graded physical activity (0–2 points; theoretical range 0–9). Group comparisons, unadjusted bivariate and adjusted multivariable regressions, false discovery rate correction, and stratified five-fold cross-validated logistic models were used. Results: Among 232 participants, 231 had a complete LCI; low, moderate, and high adherence included 84 (36.4%), 94 (40.7%), and 53 (22.9%) participants, respectively. Higher LCI scores were associated with lower adiposity, HbA1c, TyG index, triglycerides, creatinine, and SCORE2-Diabetes risk and with higher eGFR. In models adjusted for age, sex, and diabetes duration, each LCI point was associated with lower odds of obesity (aOR 0.749), elevated TyG index (0.757), albuminuria (0.716), reduced eGFR (0.802), and HbA1c ≥ 7% (0.833). Adding LCI improved cross-validated discrimination most for albuminuria (AUC 0.482 to 0.642), obesity (0.489 to 0.619), and elevated TyG index (0.524 to 0.646). The baseline albuminuria AUC was below chance, and the extended AUC remained modest. **Conclusions:** In this cross-sectional sample, higher self-reported lifestyle adherence was associated with a more favorable cardio-renal-metabolic profile. The LCI should be considered exploratory until validated against objective measures and tested in larger prospective populations.

## 1. Introduction

Type 2 diabetes mellitus (T2DM) is one of the most prevalent chronic non-communicable diseases worldwide and remains a leading cause of cardiovascular morbidity, chronic kidney disease, disability, and premature mortality. Despite major advances in pharmacological therapy, long-term metabolic control remains suboptimal for many individuals with T2DM, largely because the disease is driven by a complex interaction between biological, environmental, and behavioral factors. Consequently, contemporary diabetes management emphasizes not only pharmacological treatment but also sustained adherence to healthy lifestyle behaviors as the cornerstone of comprehensive care [1].

The International Diabetes Federation (IDF) Diabetes Atlas, 11th edition (2025), estimates that 589 million adults aged 20–79 years—approximately one in nine adults—were living with diabetes in 2024, with a projected increase to 853 million by 2050. For Romania, the same source estimates approximately 1.3 million adults with diabetes in 2024. These figures underscore the need for scalable approaches to support lifestyle management in routine diabetes care [2].

Current international guidelines consistently recommend medical nutrition therapy, regular physical activity, weight management, smoking cessation, adequate hydration, moderation of alcohol intake, and reduction in dietary sodium, saturated fats, and added sugars as essential components of diabetes management. These interventions improve insulin sensitivity, facilitate weight reduction, enhance glycemic control, reduce cardiovascular risk, and slow the progression of diabetic kidney disease. Nevertheless, achieving long-term adherence to these recommendations remains challenging in routine clinical practice, and substantial heterogeneity in lifestyle behaviors persists among adults living with T2DM [3,4,5].

The 2026 American Diabetes Association (ADA) Standards of Care grades recommendations using the ADA evidence categories (A, B, C, and E). Recommendations addressing nutrition, physical activity, weight management, and behavioral support are supported predominantly by high- or moderate-quality evidence (typically grades A or B), whereas the certainty is more variable for individual components such as hydration, alcohol, and sodium intake. Accordingly, the present LCI was framed as a pragmatic, formative behavioral summary rather than a validated psychometric scale, and its individual domains should not be interpreted as equivalent measures of exposure [6].

Although numerous studies have examined individual lifestyle factors such as physical activity, dietary quality, or body weight separately, lifestyle behaviors rarely occur in isolation. Individuals who engage in regular physical activity are also more likely to adopt healthier dietary habits, whereas poor dietary patterns frequently coexist with physical inactivity and excess adiposity. Therefore, evaluating single lifestyle components may underestimate the cumulative impact of behavioral patterns on metabolic health. Composite lifestyle scores have consequently emerged as useful tools for summarizing multiple health-related behaviors into a single quantitative measure that better reflects overall lifestyle adherence [7].

Existing composite lifestyle scores have combined combinations of smoking, alcohol, diet quality, physical activity, and adiposity, but they differ in domain definitions, weighting, and the amount of dietary information required. The proposed LCI adds a diabetes-focused, consultation-level summary that uses brief behavioral responses and a graded physical-activity component. It deliberately does not include smoking cessation as a separate point because smoking status was collected for descriptive and confounding purposes, while the index was restricted to daily behaviors that could be assessed as current adherence during the visit. This design choice is exploratory and limits the scope of the index [8,9,10].

Several lifestyle indices have previously been proposed in epidemiological research, including healthy lifestyle scores derived from diet quality, smoking status, alcohol consumption, physical activity, and body weight. However, most available indices were developed for general populations rather than specifically for individuals with T2DM. Furthermore, many require extensive dietary questionnaires or detailed nutritional assessments that are difficult to implement during routine outpatient consultations. There remains a need for a simple, clinically applicable lifestyle assessment tool that incorporates routinely collected behavioral information while maintaining adequate discriminatory ability for clinically relevant metabolic outcomes [8,11].

Lifestyle behaviors are particularly relevant because they influence multiple interconnected components of the cardio-renal-metabolic continuum. Poor dietary quality, sedentary behavior, and excess caloric intake contribute to obesity, insulin resistance, dyslipidemia, hypertension, systemic inflammation, and progressive renal dysfunction, all of which increase cardiovascular risk in patients with T2DM. Conversely, sustained adherence to healthy lifestyle recommendations has been associated with improvements in glycated hemoglobin, triglyceride concentrations, insulin sensitivity, blood pressure, body composition, and renal function. These observations suggest that comprehensive lifestyle adherence should be evaluated as an integrated construct rather than as a collection of independent behaviors [12,13].

Among routinely available metabolic biomarkers, the triglyceride-glucose (TyG) index has emerged as a practical surrogate marker of insulin resistance and has been associated with obesity, metabolic syndrome, non-alcoholic fatty liver disease, chronic kidney disease, and cardiovascular events. Similarly, glycated hemoglobin (HbA1c), estimated glomerular filtration rate (eGFR), urinary albumin-to-creatinine ratio (UACR), and SCORE2-Diabetes provide complementary information regarding glycemic control, renal involvement, and cardiovascular risk. Evaluating these biomarkers together may provide a more comprehensive understanding of how lifestyle adherence relates to the overall cardio-renal-metabolic phenotype [14].

Despite growing evidence supporting lifestyle modification in T2DM, relatively few studies have investigated whether a simple composite lifestyle score can provide incremental clinical information beyond conventional demographic characteristics. Moreover, the potential ability of such an index to identify patients with obesity, insulin resistance, albuminuria, impaired renal function, or suboptimal glycemic control has not been sufficiently explored in real-world outpatient populations [9,15].

Therefore, the present study aimed to develop a pragmatic Lifestyle Compliance Index (LCI) based exclusively on routinely collected lifestyle variables and to evaluate its relationship with metabolic, renal, and cardiovascular health in adults with T2DM. Specifically, the objectives were to (I) characterize lifestyle behaviors within the study population, (II) develop and describe the distribution of the Lifestyle Compliance Index, (III) compare metabolic and renal characteristics according to lifestyle adherence categories, (IV) investigate the associations between LCI and cardio-renal-metabolic biomarkers, and (V) evaluate the incremental discriminative value of the LCI for identifying clinically relevant metabolic and renal abnormalities. We hypothesized that greater adherence to recommended lifestyle behaviors would be associated with a more favorable cardio-renal-metabolic profile and would provide additional information beyond basic demographic characteristics in the identification of individuals with adverse metabolic outcomes.

## 2. Materials and Methods

### 2.1. Study Design and Population

This cross-sectional observational study used a real-world clinical database of adults with type 2 diabetes mellitus (T2DM) attending a specialized outpatient diabetes clinic in Romania. Records were collected during routine consultations between 1 October 2024 and 19 February 2026, prospectively entered into the clinical database, and anonymized before analysis. The database was examined retrospectively to evaluate associations between self-reported lifestyle adherence and cardio-renal-metabolic health. The study was reported according to the STROBE guidance for cross-sectional studies..

All 232 consecutive records meeting the study eligibility criteria during the recruitment period were retained. Eligible patients were adults (≥18 years) with confirmed T2DM and complete essential demographic, anthropometric, biochemical, cardiovascular, renal, and lifestyle information in the electronic medical record. Patients with type 1 diabetes, gestational diabetes, secondary diabetes, or missing essential data were excluded. The source database did not record the total number of patients approached, the number assessed for eligibility, or the number excluded before database entry; therefore, the selection fraction and non-response rate could not be estimated and are acknowledged as potential sources of selection bias.

### 2.2. Clinical, Anthropometric, and Laboratory Assessment

Demographic information included age, sex, place of residence, educational level, smoking status, family history of diabetes and cardiovascular disease, and diabetes duration. Anthropometric assessment comprised body weight, height, BMI, and waist circumference. Blood pressure was recorded during the outpatient clinical visit with the patient seated and supported according to routine clinic practice, and the recorded systolic and diastolic values were retained. Because the retrospective database stored only the final values, it did not capture the measurement arm, cuff dimensions, device manufacturer/model or validation certificate, number and interval of repeated readings, or whether an average of repeated measurements was used. These unrecorded protocol elements limit reproducibility and are explicitly acknowledged as a measurement limitation.

Clinical comorbidities extracted from the database included hypertension, dyslipidemia, obesity, chronic kidney disease, myocardial infarction, coronary revascularization, stroke, peripheral arterial disease, heart failure, atrial fibrillation, chronic venous insufficiency, hepatic steatosis, thyroid disease, chronic pulmonary disease, psychiatric disorders, and malignant diseases.

Laboratory evaluation included fasting plasma glucose, glycated hemoglobin (HbA1c), complete blood count, lipid profile, serum creatinine, serum urea, estimated glomerular filtration rate (eGFR), urinary albumin-to-creatinine ratio (UACR), liver enzymes, C-reactive protein (CRP), and additional biochemical parameters routinely measured during diabetes follow-up. The triglyceride-glucose (TyG) index and Fibrosis-4 (FIB-4) score were calculated according to their original published equations. Ten-year cardiovascular risk was estimated using the SCORE2-Diabetes algorithm according to the recommendations of the European Society of Cardiology.

### 2.3. Lifestyle Assessment and Development of the Lifestyle Compliance Index

Lifestyle information was obtained with a standardized questionnaire completed during routine clinical evaluation. The eight domains were selected a priori from guideline-supported behaviors relevant to adults with T2DM: hydration, fruit and vegetable intake, sweets, saturated fat, sugar-sweetened carbonated beverages, alcohol, dietary sodium, and physical activity. ‘Adequate daily hydration’ was coded as yes when the patient reported meeting the clinic’s recommended daily fluid intake; the questionnaire did not record volume, beverage composition, or an objective verification measure. ‘Daily fruit and vegetable consumption’ was coded as yes when the patient reported consuming fruit and vegetables every day; portion sizes, specific types, and preparation methods were not captured. Thus, these variables represent self-reported adherence indicators rather than validated quantitative dietary measurements.

For construction of the LCI, each healthy behavior contributed one point. Physical activity was graded using the recorded International Physical Activity Questionnaire category: inactive = 0, minimally active = 1, and health-enhancing physical activity (HEPA) = 2. This grading reflects the ordered exposure information available for physical activity and does not imply that physical activity is causally twice as important as any other domain. The theoretical score ranged from 0 to 9. One invalid saturated-fat response (‘fa’) prevented calculation of a complete LCI and was treated as missing, leaving 231 participants for LCI-based analyses. Cronbach’s alpha was not calculated because the domains are formative, non-interchangeable behaviors rather than reflective items; test–retest reliability and criterion validity against accelerometry, dietary records, or an established lifestyle instrument were not available.

For descriptive stratification only, the observed score distribution was grouped into low adherence (1–4 points), moderate adherence (5–6 points), and high adherence (7–9 points). These cut-offs were pragmatic, data-driven intervals chosen to avoid sparse extreme cells and were not predefined clinical thresholds. All primary association and discrimination analyses therefore treated LCI as a continuous variable; the categories should not be interpreted as validated severity classes.

### 2.4. Outcome Measures

The primary objective of the study was to evaluate the relationship between lifestyle adherence and cardio-renal-metabolic health in adults with T2DM. Secondary objectives included comparison of anthropometric, glycemic, lipid, renal, inflammatory, and cardiovascular characteristics across Lifestyle Compliance Index categories; assessment of correlations between the continuous LCI score and metabolic biomarkers; and evaluation of the incremental discriminative value of the LCI for clinically relevant metabolic and renal abnormalities.

Because the original retrospective database did not have a registered prospective protocol, no single primary outcome had been prespecified before analysis. For transparent reporting, HbA1c was designated as the principal metabolic outcome for the sample-size sensitivity analysis; all other biomarkers and binary endpoints were treated as secondary or exploratory outcomes. Outcome definitions were based on the 2026 ADA Standards of Care for glycemic control and obesity-related thresholds, the ADA–KDIGO consensus framework (2022) and KDIGO chronic kidney disease definitions for eGFR and albuminuria, and previously published literature for the TyG threshold (>9.04). The binary outcomes were poor glycemic control (HbA1c ≥ 7.0%), elevated TyG index (>9.04), obesity (BMI ≥ 30 kg/m^2^), reduced renal function (eGFR < 60 mL/min/1.73 m^2^), and albuminuria (UACR ≥ 30 mg/g).

### 2.5. Statistical Analysis

Continuous variables were assessed for normality using the Shapiro–Wilk test together with graphical inspection of histograms and quantile–quantile plots. Variables with approximately normal distributions are presented as mean ± standard deviation, whereas non-normally distributed variables are reported as median and interquartile range. Categorical variables are presented as absolute frequencies and percentages.

Comparisons among the three LCI categories used one-way ANOVA for approximately normal continuous variables or the Kruskal–Wallis test for skewed variables. Significant omnibus ANOVA results were followed by Tukey’s honestly significant difference test; significant Kruskal–Wallis results were followed by pairwise Mann–Whitney U tests with Holm adjustment. Categorical variables were compared using Pearson’s chi-square test or Fisher’s exact test when expected cell counts were small.

Spearman correlations were retained as descriptive sensitivity analyses, with Benjamini–Hochberg false discovery rate correction across the 14 biomarker comparisons. To directly compare model-based estimates, unadjusted bivariate linear or logistic regression models were fitted first, followed by prespecified multivariable models adjusted for age, sex, and diabetes duration. Medication-adjusted sensitivity models additionally included indicators for any glucose-lowering, antihypertensive, and lipid-lowering medication. Continuous outcomes and LCI were standardized for linear models. Linear-model assumptions were evaluated using residual-versus-fitted and Q–Q plots and heteroscedasticity checks; multicollinearity was assessed with variance-inflation factors (all VIFs 1.06–1.28 in the medication-adjusted covariate set). Logistic models were assessed using discrimination, Brier score, and calibration diagnostics.

Because this was a retrospective analysis of all eligible records in the source database, no prospective sample-size calculation or allowance for non-response/missing data had been performed before data collection. For transparency, a post hoc sensitivity calculation using 231 LCI-complete participants and three adherence groups indicated approximately 80% power at a two-sided α = 0.05 to detect a one-way ANOVA effect size of f = 0.206 or larger. For a one-predictor regression coefficient tested with three adjustment covariates, the corresponding detectable effect was approximately Cohen’s f^2^ = 0.034. These calculations indicate limited sensitivity to small effects, particularly in the smallest high-adherence subgroup (n = 53), and the findings should be interpreted as exploratory.

Medication indicators were derived from the active-treatment fields in the database. Any glucose-lowering treatment included oral agents, injectable agents, insulin, or diet therapy; antihypertensive treatment included the recorded antihypertensive classes; lipid-lowering treatment included statins, ezetimibe, fibrates, omega-3 therapy, or PCSK9-directed therapy. These variables were not included in the primary demographic-only models because treatment intensity may partly reflect disease severity and may lie on the pathway between lifestyle and measured control; they were included in prespecified sensitivity models to quantify residual confounding.

### 2.6. Incremental Discriminative Analysis

To evaluate incremental discrimination, baseline logistic regression models including age, sex, and diabetes duration were compared with extended models additionally incorporating continuous LCI. Stratified five-fold cross-validation generated out-of-fold predictions. ROC AUC, average precision, Brier score, and paired-bootstrap confidence intervals for the change in AUC were reported. These models assessed discrimination of prevalent abnormalities in cross-sectional data and were not interpreted as predictions of future events or clinical utility.

Model performance was summarized with ROC AUC, average precision, and Brier score; paired bootstrap resampling of out-of-fold predictions provided confidence intervals for changes in AUC. These metrics describe discrimination of prevalent abnormalities and were not interpreted as prospective predictions.

### 2.7. Data Quality

Prior to statistical analysis, all variables underwent systematic quality assessment. Continuous variables were screened for biologically implausible values and inconsistencies. One HbA1c value of 114% was considered biologically impossible and was treated as missing in all analyses involving glycated hemoglobin. Additionally, one participant provided an invalid response regarding saturated fat intake (“fa”), preventing calculation of the complete Lifestyle Compliance Index; this observation was therefore excluded only from analyses requiring the LCI. No additional implausible values requiring exclusion were identified. Missing values were handled using complete-case analysis for descriptive and inferential statistics, while regression analyses included all participants with complete information for the respective variables.

### 2.8. Ethical Considerations

The study was conducted in accordance with the ethical principles of the Declaration of Helsinki. All clinical data were anonymized before statistical analysis, and no personally identifiable information was accessible to the investigators. The study protocol was approved by the appropriate Institutional Ethics Committee (Approval No. 42/31 October 2025). Because only anonymized routinely collected clinical data were analyzed retrospectively, the requirement for written informed consent was waived according to institutional regulations.

## 3. Results

### 3.1. Lifestyle Characteristics of the Study Population

Among the 232 participants, complete LCI scores were available for 231 because one saturated-fat response was invalid. The recruitment period, baseline characteristics, lifestyle behaviors, laboratory values, and medication distribution across LCI categories are summarized in Table 1.

Data are presented as mean ± standard deviation, median (interquartile range), or n (%), as appropriate. Overall values use all available records; LCI-category columns use the 231 complete LCI records. Group comparisons used one-way ANOVA or Kruskal–Wallis tests for continuous variables and Pearson’s chi-square or Fisher’s exact test for categorical variables. Missing values are reported in the penultimate column.

Adequate daily hydration was reported by 163 participants (70.3%), whereas 69 individuals (29.7%) did not achieve the recommended fluid intake. Regular daily consumption of fruits and vegetables was reported by 171 participants (73.7%), while 61 (26.3%) indicated that they did not consume these foods on a daily basis.

With regard to dietary habits, 143 participants (61.6%) reported limiting the consumption of sweets, whereas 89 (38.4%) did not. In contrast, adherence to recommendations regarding saturated fat intake was considerably lower. Only 97 participants (42.0%) reported reducing saturated fat consumption, while 134 (58.0%) did not; one questionnaire contained an invalid response and was excluded from this specific variable.

Avoidance of sugar-sweetened carbonated beverages represented the most frequently adopted healthy behavior. A total of 184 participants (79.3%) reported limiting carbonated soft drinks, whereas 48 (20.7%) continued to consume them regularly. Alcohol consumption was reported by 51 participants (22.0%), while 181 (78.0%) declared no alcohol intake.

Salt restriction was less frequently observed. Only 98 participants (42.2%) reported limiting salt intake to less than 5 g/day, whereas 134 (57.8%) exceeded this recommendation.

Physical activity levels were generally low. According to the International Physical Activity Questionnaire categorization, 134 participants (57.8%) were classified as inactive, 67 (28.9%) as minimally active, and only 31 (13.4%) achieved health-enhancing physical activity (HEPA) levels.

These distributions are summarized in Table 2; the complete baseline, lifestyle, laboratory, and treatment profile is provided in Table 1.

### 3.2. Development and Distribution of the Lifestyle Compliance Index

The LCI incorporated eight domains and ranged theoretically from 0 to 9 points. The observed mean was 5.03 ± 1.80, median 5 (IQR 4–6), and observed range 1–9.

Among the 231 LCI-complete participants, low adherence included 84 (36.4%), moderate adherence 94 (40.7%), and high adherence 53 (22.9%).

The category counts are descriptive and reflect the observed score distribution; no clinical threshold is implied.

**Figure 1:**
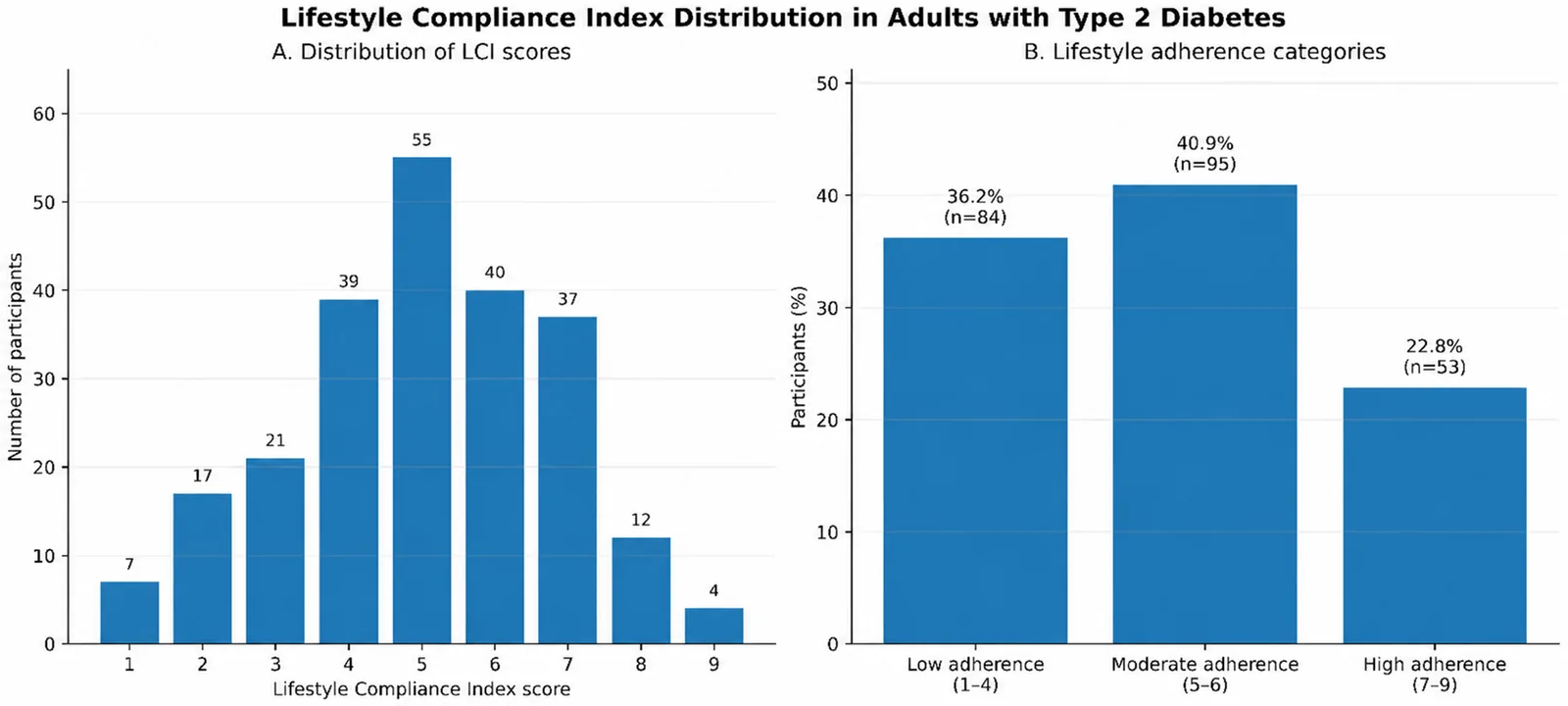
Distribution of the Lifestyle Compliance Index and Adherence Categories in Adults with Type 2 Diabetes. Panel (**A**) shows the distribution of the Lifestyle Compliance Index (LCI) scores in the study population. The LCI incorporated adequate hydration, daily fruit and vegetable consumption, reduced intake of sweets, saturated fats and carbonated beverages, salt intake below 5 g/day, abstinence from alcohol, and physical activity level. Scores ranged from 1 to 9, with a mean of 5.03 ± 1.80 and a median of 5 points (IQR: 4–6). Panel (**B**) presents the predefined adherence categories: low adherence (1–4 points), moderate adherence (5–6 points), and high adherence (7–9 points). Values above the bars represent absolute frequencies or percentages, as appropriate.

Table 3 and Figure 1 show the score distribution and descriptive categories.

The LCI distribution and descriptive adherence categories are shown in Table 3 and Figure 1. These categories summarize the observed score distribution and are not validated clinical classes.

### 3.3. Metabolic and Renal Characteristics According to Lifestyle Compliance

Participants with complete LCI data were compared across low (1–4 points; n = 84), moderate (5–6 points; n = 94), and high (7–9 points; n = 53) adherence categories. Baseline age and diabetes duration were similar across groups (Table 4).

A progressively more favorable anthropometric profile was observed with increasing lifestyle adherence. Mean body mass index decreased from 32.98 ± 6.38 kg/m^2^ in the low-adherence group to 30.71 ± 5.22 kg/m^2^ in the moderate-adherence group and 29.28 ± 4.56 kg/m^2^ in the high-adherence group (*p* < 0.001). Post hoc analysis showed significantly lower BMI in the moderate-adherence group compared with the low-adherence group (adjusted *p* = 0.018) and in the high-adherence group compared with the low-adherence group (adjusted *p* < 0.001).

Waist circumference also declined progressively across the three categories, from 111.55 ± 14.43 cm in the low-adherence group to 105.22 ± 13.50 cm in the moderate-adherence group and 99.19 ± 10.48 cm in the high-adherence group (*p* < 0.001). All pairwise comparisons remained statistically significant after correction for multiple testing. Correspondingly, the prevalence of obesity decreased from 65.5% in the low-adherence group to 52.6% in the moderate-adherence group and 41.5% in the high-adherence group (*p* = 0.020).

Systolic blood pressure was also lower among participants with greater lifestyle adherence. Mean systolic blood pressure decreased from 150.29 ± 20.64 mmHg in the low-adherence group to 144.72 ± 19.84 mmHg in the moderate-adherence group and 140.94 ± 20.35 mmHg in the high-adherence group (*p* = 0.026). The significant post hoc difference was observed between the low- and high-adherence groups (adjusted *p* = 0.025). Diastolic blood pressure and the prevalence of physician-diagnosed hypertension did not differ significantly among the groups.

Glycemic parameters showed a generally favorable pattern with increasing adherence. Median HbA1c was 7.40% (IQR: 6.50–8.95%) in the low-adherence group, 7.10% (IQR: 6.30–8.57%) in the moderate-adherence group, and 6.50% (IQR: 6.10–7.80%) in the high-adherence group (*p* = 0.025). The proportion of participants with HbA1c ≥ 7% was 57.1%, 53.2%, and 37.7%, respectively; the global categorical comparison was not statistically significant (*p* = 0.075). Fasting plasma glucose did not differ significantly among categories (*p* = 0.184).

The TyG index decreased from 9.36 (IQR: 9.02–10.00) in the low-adherence group to 9.12 (IQR: 8.71–9.52) in the moderate-adherence group and 8.94 (IQR: 8.67–9.43) in the high-adherence group (*p* < 0.001). The prevalence of a TyG index > 9.04 was 73.8%, 53.2%, and 45.3%, respectively (*p* = 0.001).

Median triglyceride concentration decreased from 169 mg/dL (IQR: 110–259.5 mg/dL) in the low-adherence category to 119.5 mg/dL (IQR: 92–174.25 mg/dL) and 117 mg/dL (IQR: 83–153 mg/dL) in the moderate- and high-adherence categories, respectively (*p* < 0.001). HDL cholesterol increased from 39 mg/dL to 41 mg/dL and 45 mg/dL across the three groups (*p* = 0.005), whereas total and LDL cholesterol did not differ significantly.

Renal parameters were also more favorable among participants with higher LCI values. Median serum creatinine decreased from 0.88 mg/dL (IQR: 0.73–1.09 mg/dL) in the low-adherence group to 0.85 mg/dL (IQR: 0.66–0.97 mg/dL) in the moderate-adherence group and 0.75 mg/dL (IQR: 0.64–0.91 mg/dL) in the high-adherence group (*p* = 0.007). Median eGFR increased correspondingly from 73.69 mL/min/1.73 m^2^ (IQR: 52.56–91.60) to 84.39 mL/min/1.73 m^2^ (IQR: 61.54–94.92) and 89.00 mL/min/1.73 m^2^ (IQR: 73.46–98.71), respectively (*p* = 0.007). For both variables, the significant pairwise difference was observed between the low- and high-adherence groups.

Albuminuria prevalence decreased across categories (50.0%, 28.7%, and 20.8%; *p* < 0.001), whereas continuous UACR did not differ significantly (*p* = 0.115). This pattern is compatible with the influence of skewness, biological variability, and dichotomization of a continuous measure and should not be interpreted as evidence of a causal renal effect.

Mean SCORE2-Diabetes risk was 36.96 ± 16.84%, 35.71 ± 14.32%, and 30.04 ± 15.64% in the low-, moderate-, and high-adherence groups, respectively (*p* = 0.034).

**Figure 2:**
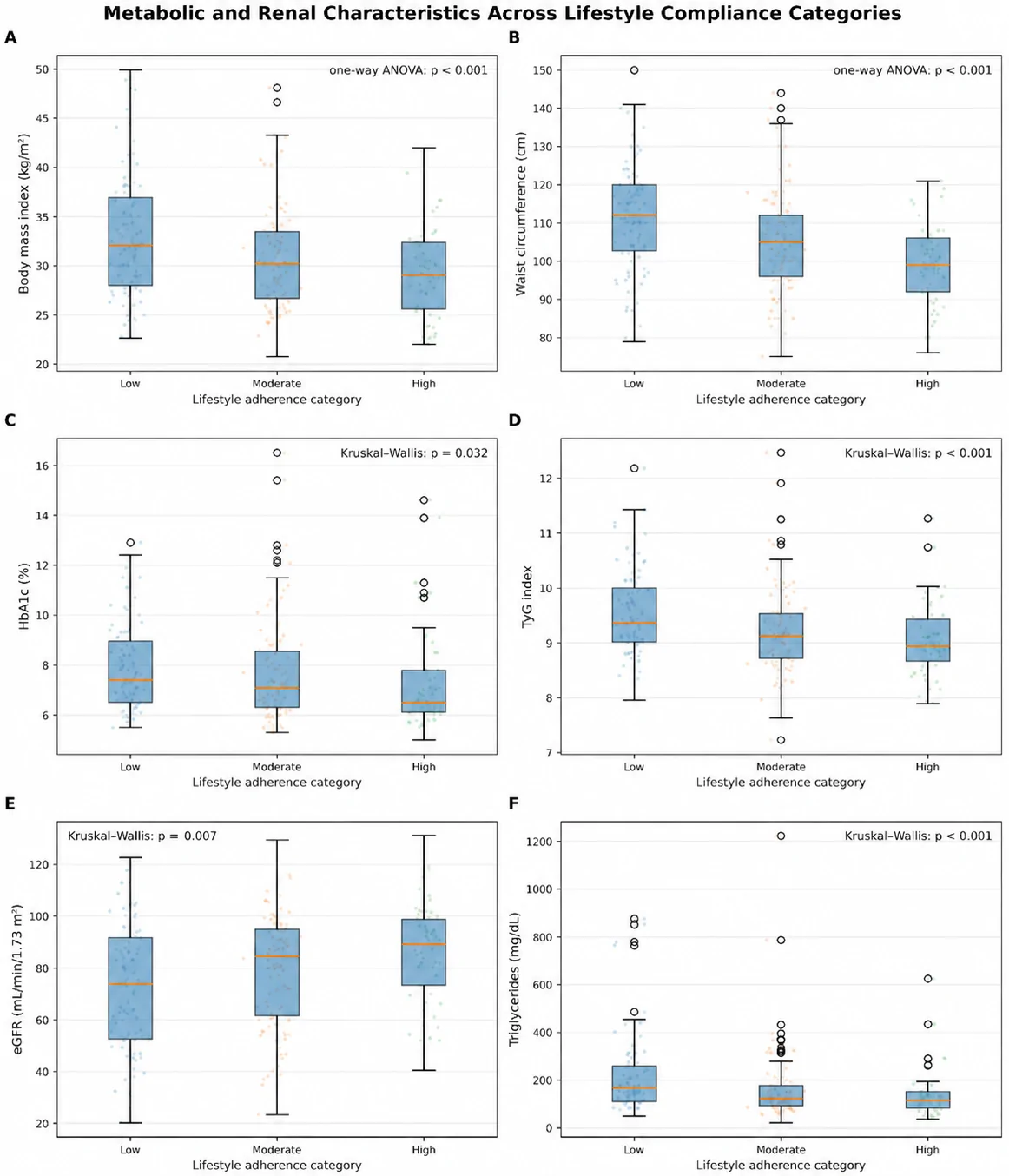
Metabolic and renal characteristics across lifestyle compliance categories. (**A**) Body mass index (kg/m²); (**B**) waist circumference (cm); (**C**) glycated hemoglobin (HbA1c, %); (**D**) triglyceride–glucose (TyG) index; (**E**) estimated glomerular filtration rate (eGFR, mL/min/1.73 m²); (**F**) triglyceride concentration (mg/dL). Boxes represent the interquartile range, horizontal orange lines indicate the median, and whiskers extend to the most extreme observations within 1.5 times the interquartile range. Semi-transparent colored circles represent individual participant observations, whereas open black circles indicate observations beyond the whiskers (outliers). Overall group-comparison *p*-values were calculated using one-way ANOVA for panels (**A**,**B**) and the Kruskal–Wallis test for panels (**C**–**F**).

Group-specific estimates, missing observations, and the statistical tests used are reported in Table 4 and Figure 2.

### 3.4. Correlations and Adjusted Associations Between Lifestyle Compliance and Metabolic Control

Unadjusted bivariate regression estimates were compared directly with prespecified multivariable estimates adjusted for age, sex, and diabetes duration; Spearman coefficients were retained as descriptive sensitivity measures (Table 5).

Higher LCI scores were associated with a more favorable anthropometric profile. The strongest correlation was observed with waist circumference (ρ = −0.362, FDR-adjusted *p* < 0.001), followed by body mass index (ρ = −0.252, FDR-adjusted *p* < 0.001). Lifestyle compliance was also inversely associated with systolic blood pressure (ρ = −0.185, FDR-adjusted *p* = 0.008), whereas no significant relationship was observed with diastolic blood pressure.

Glycemic control improved modestly with increasing lifestyle adherence. The LCI was inversely correlated with fasting plasma glucose (ρ = −0.175, FDR-adjusted *p* = 0.011) and HbA1c (ρ = −0.199, FDR-adjusted *p* = 0.005). The strongest metabolic association was observed for the TyG index (ρ = −0.294, FDR-adjusted *p* < 0.001). Triglyceride concentrations were also inversely correlated with LCI (ρ = −0.256, FDR-adjusted *p* < 0.001), while HDL cholesterol showed a positive correlation (ρ = 0.200, FDR-adjusted *p* = 0.005).

Higher lifestyle adherence was also associated with a more favorable renal profile. LCI correlated inversely with serum creatinine (ρ = −0.196, FDR-adjusted *p* = 0.005) and positively with estimated glomerular filtration rate (ρ = 0.194, FDR-adjusted *p* = 0.005). Weak inverse correlations were observed with urinary albumin-to-creatinine ratio (ρ = −0.131, FDR-adjusted *p* = 0.050) and C-reactive protein (ρ = −0.146, FDR-adjusted *p* = 0.031). SCORE2-Diabetes was also weakly inversely correlated with LCI (ρ = −0.170, FDR-adjusted *p* = 0.012).

Multivariable linear regression analyses were subsequently performed to determine whether these relationships remained after adjustment for age, sex, and diabetes duration. All continuous outcomes and the LCI were standardized before modelling, allowing the regression coefficients to represent the standard-deviation change in each outcome associated with a one-standard-deviation increase in lifestyle compliance.

After adjustment, higher LCI remained independently associated with lower body mass index (standardized β = −0.267, 95% CI: −0.394 to −0.139; *p* < 0.001) and lower waist circumference (β = −0.324, 95% CI: −0.439 to −0.210; *p* < 0.001). Higher adherence was also associated with lower systolic blood pressure (β = −0.188, 95% CI: −0.320 to −0.055; *p* = 0.005).

The adjusted associations with metabolic control also remained statistically significant. A higher LCI was associated with lower HbA1c (β = −0.158, 95% CI: −0.290 to −0.027; *p* = 0.018), lower TyG index (β = −0.291, 95% CI: −0.416 to −0.167; *p* < 0.001), and lower triglyceride concentrations (β = −0.188, 95% CI: −0.308 to −0.069; *p* = 0.002). The positive unadjusted association between LCI and HDL cholesterol was attenuated after adjustment and was no longer statistically significant (β = 0.130, *p* = 0.143).

Renal associations also persisted in the adjusted models. Higher lifestyle compliance was associated with lower serum creatinine (β = −0.144, 95% CI: −0.248 to −0.039; *p* = 0.007) and higher eGFR (β = 0.160, 95% CI: 0.041–0.278; *p* = 0.008). LCI remained inversely associated with SCORE2-Diabetes after adjustment for age, sex, and diabetes duration (β = −0.157, 95% CI: −0.240 to −0.074; *p* < 0.001).

Adjusted logistic regression analyses were additionally performed for clinically relevant binary outcomes. Each one-point increase in LCI was associated with 25.1% lower odds of obesity (adjusted odds ratio [aOR] = 0.749, 95% CI: 0.639–0.878; *p* < 0.001). Similarly, each additional LCI point was associated with lower odds of HbA1c ≥ 7% (aOR = 0.833, 95% CI: 0.710–0.976; *p* = 0.024), TyG index > 9.04 (aOR = 0.757, 95% CI: 0.645–0.889; *p* < 0.001), eGFR < 60 mL/min/1.73 m^2^ (aOR = 0.802, 95% CI: 0.672–0.958; *p* = 0.015), and albuminuria ≥ 30 mg/g (aOR = 0.716, 95% CI: 0.606–0.845; *p* < 0.001).

In unadjusted bivariate models, standardized β coefficients were −0.275 for BMI, −0.351 for waist circumference, −0.159 for HbA1c, −0.280 for TyG index, −0.176 for triglycerides, and 0.196 for eGFR. The corresponding unadjusted odds ratios per LCI point were 0.750 for obesity, 0.849 for HbA1c ≥ 7%, 0.767 for TyG index > 9.04, 0.787 for eGFR < 60 mL/min/1.73 m^2^, and 0.729 for albuminuria ≥ 30 mg/g. Adjustment attenuated some estimates but did not materially change the direction of association.

In medication-adjusted sensitivity models, associations were materially similar: aORs were 0.754 for obesity, 0.820 for HbA1c ≥ 7%, 0.739 for TyG index > 9.04, 0.812 for eGFR < 60 mL/min/1.73 m^2^, and 0.715 for albuminuria ≥ 30 mg/g. Standardized β coefficients were −0.260 for BMI, −0.302 for waist circumference, −0.158 for HbA1c, −0.286 for TyG, −0.190 for triglycerides, −0.138 for creatinine, 0.151 for eGFR, and −0.151 for SCORE2-Diabetes; the HDL association was attenuated (β = 0.123, *p* = 0.060) (Table 6).

Overall, higher LCI values were associated with more favorable anthropometric, glycemic, lipid, renal, and cardiovascular-risk measures. The estimates are cross-sectional associations and remain vulnerable to residual confounding and reverse causality.

**Table 5:** Spearman Correlations Between the Lifestyle Compliance Index and Clinical/Metabolic Parameters.

Parameter	n	Spearman ρ	Unadjusted *p* Value	FDR-Adjusted *p* Value
Waist circumference	231	−0.362	<0.001	<0.001
TyG index	231	−0.294	<0.001	<0.001
Triglycerides	231	−0.256	<0.001	<0.001
Body mass index	231	−0.252	<0.001	<0.001
HDL cholesterol	231	0.200	0.002	0.005
HbA1c	230	−0.199	0.002	0.005
Serum creatinine	231	−0.196	0.003	0.005
Estimated glomerular filtration rate	231	0.194	0.003	0.005
Systolic blood pressure	231	−0.185	0.005	0.008
Fasting plasma glucose	231	−0.175	0.008	0.011
SCORE2-Diabetes	231	−0.170	0.010	0.012
C-reactive protein	231	−0.146	0.027	0.031
Urinary albumin-to-creatinine ratio	231	−0.131	0.046	0.050
Diastolic blood pressure	231	−0.085	0.200	0.200

Correlations were calculated using Spearman’s rank correlation coefficient. *p* values were adjusted across the 14 comparisons using the Benjamini–Hochberg false discovery rate procedure. HbA1c was available for 230 participants in the correlation analysis because one additional clinically implausible HbA1c value was treated as missing.

**Table 6:** Adjusted Associations Between the Lifestyle Compliance Index and Clinically Relevant Outcomes.

Outcome	Adjusted Effect per LCI Point	95% CI	*p* Value
Obesity	aOR 0.749	0.639–0.878	<0.001
HbA1c ≥ 7%	aOR 0.833	0.710–0.976	0.024
TyG index > 9.04	aOR 0.757	0.645–0.889	<0.001
eGFR < 60 mL/min/1.73 m^2^	aOR 0.802	0.672–0.958	0.015
Albuminuria ≥ 30 mg/g	aOR 0.716	0.606–0.845	<0.001

Adjusted odds ratios were estimated using binary logistic regression models including age, sex, and diabetes duration as covariates. An aOR below 1 indicates lower odds of the respective outcome for each one-point increase in the Lifestyle Compliance Index. Because the data were cross-sectional, the results represent adjusted associations rather than prospective risk reductions. Abbreviations: aOR, adjusted odds ratio; CI, confidence interval; C-reactive protein; eGFR, estimated glomerular filtration rate; FDR, false discovery rate; HbA1c, glycated hemoglobin; LCI, Lifestyle Compliance Index; SCORE2-Diabetes, Systematic Coronary Risk Evaluation 2–Diabetes; TyG, triglyceride-glucose; UACR, urinary albumin-to-creatinine ratio.

**Figure 3:**
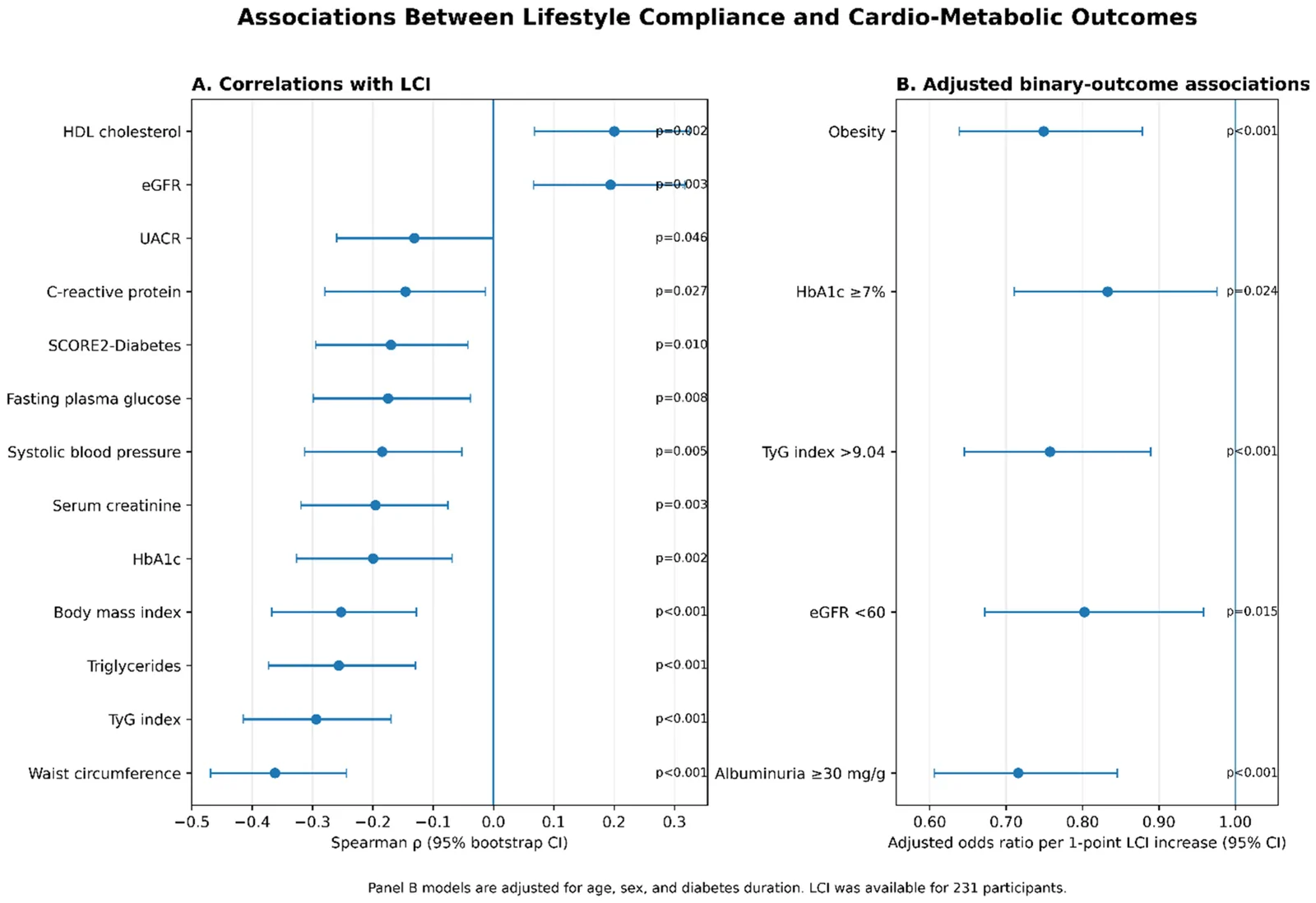
Correlations and Adjusted Associations Between the Lifestyle Compliance Index and Cardio-Metabolic Outcomes. Panel (**A**) presents Spearman correlation coefficients and 95% bootstrap confidence intervals for the associations between the Lifestyle Compliance Index (LCI) and anthropometric, glycemic, lipid, renal, inflammatory, and cardiovascular-risk parameters. Negative coefficients indicate that higher lifestyle adherence was associated with lower values of the respective parameter, whereas positive coefficients indicate direct associations. Panel (**B**) presents adjusted odds ratios and 95% confidence intervals for clinically relevant binary outcomes per one-point increase in LCI. Logistic regression models were adjusted for age, sex, and diabetes duration. The vertical reference lines indicate no correlation (ρ = 0) in Panel (**A**) and no association (adjusted odds ratio = 1) in Panel (**B**). LCI was available for 231 participants; HbA1c-related analyses included 230 participants after exclusion of one clinically implausible value.

Figure 3 presents the correlations and adjusted associations between the lifestyle compliance index and cardio-metabolic outcomes.

### 3.5. Incremental Discriminative Value of the Lifestyle Compliance Index

For each outcome, the baseline model included age, sex, and diabetes duration, and the extended model added continuous LCI. Model performance was evaluated using stratified five-fold cross-validated out-of-fold predictions. The baseline AUC of 0.482 for albuminuria was below chance, so the increase to 0.642 should be interpreted as improvement over a weak baseline rather than evidence of strong clinical discrimination.

The LCI was available for 231 participants after exclusion of one participant with an invalid response for saturated fat intake. HbA1c-related analyses included 230 participants because one additional clinically implausible HbA1c value was treated as missing. For each outcome, a baseline logistic regression model incorporating age, sex, and diabetes duration was compared with an extended model that additionally included the LCI. Model performance was evaluated using stratified five-fold cross-validated out-of-fold predictions.

Higher lifestyle compliance remained significantly associated with lower odds of all five evaluated abnormalities after adjustment for age, sex, and diabetes duration. Each one-point increase in LCI was associated with lower odds of HbA1c ≥ 7% (adjusted odds ratio [aOR] = 0.833, 95% CI: 0.710–0.976; *p* = 0.024), TyG index > 9.04 (aOR = 0.757, 95% CI: 0.645–0.889; *p* < 0.001), obesity (aOR = 0.749, 95% CI: 0.639–0.878; *p* < 0.001), eGFR < 60 mL/min/1.73 m^2^ (aOR = 0.802, 95% CI: 0.672–0.958; *p* = 0.015), and albuminuria ≥ 30 mg/g (aOR = 0.716, 95% CI: 0.606–0.845; *p* < 0.001).

The addition of LCI to age, sex, and diabetes duration improved cross-validated discrimination for each evaluated outcome, although the magnitude and statistical robustness of the improvement varied. For HbA1c ≥ 7%, the ROC AUC increased from 0.660 in the baseline model to 0.688 after inclusion of LCI. The corresponding increase of 0.028 was modest and its bootstrap confidence interval included zero (95% CI: −0.008 to 0.064; bootstrap *p* = 0.130). Average precision remained nearly unchanged, increasing from 0.675 to 0.676.

For elevated TyG index, addition of LCI produced a larger improvement. The ROC AUC increased from 0.524 to 0.646, corresponding to an absolute gain of 0.122 (95% bootstrap CI: 0.043–0.203; *p* = 0.001). Average precision increased from 0.607 to 0.704, while the Brier score decreased from 0.243 to 0.232, indicating improved probabilistic accuracy.

A similar pattern was observed for obesity. The baseline model showed no meaningful discrimination, with a ROC AUC of 0.489. After inclusion of the LCI, the ROC AUC increased to 0.619, representing an absolute improvement of 0.129 (95% CI: 0.052–0.203; *p* < 0.001). Average precision increased from 0.539 to 0.648, and the Brier score decreased from 0.254 to 0.240.

For reduced renal function, the ROC AUC increased from 0.641 to 0.673 after adding LCI. However, the improvement of 0.033 was not statistically robust in bootstrap analysis (95% CI: −0.013 to 0.077; *p* = 0.163). Average precision increased modestly from 0.365 to 0.379, while the Brier score changed from 0.183 to 0.180.

The largest absolute improvement was observed for albuminuria (AUC 0.482 to 0.642; ΔAUC 0.160, 95% CI 0.063–0.251; *p* = 0.002). Because the baseline model performed below chance and the extended AUC remained modest, this result does not establish clinical utility.

LCI contributed the most incremental information for albuminuria, obesity, and elevated TyG index, whereas gains for HbA1c ≥ 7% and reduced eGFR were small and not statistically robust. These are exploratory discriminative findings rather than validated diagnostic performance.

The LCI should not be used as a diagnostic instrument or as a replacement for biochemical, anthropometric, or renal assessment. Its potential role is limited to brief behavioral profiling pending validation (Table 7 and Table 8).

**Table 7:** Incremental Cross-Validated Performance of the Lifestyle Compliance Index for Metabolic and Renal Abnormalities.

Outcome	Events, n/N	Baseline Model ROC AUC	Baseline + LCI ROC AUC	ΔAUC (95% Bootstrap CI)	Baseline AP	Baseline + LCI AP	Baseline Brier Score
HbA1c ≥ 7%	118/230	0.660	0.688	0.028 (−0.008 to 0.064)	0.675	0.676	0.229
TyG index > 9.04	136/231	0.524	0.646	0.122 (0.043–0.203)	0.607	0.704	0.243
Obesity	126/231	0.489	0.619	0.129 (0.052–0.203)	0.539	0.648	0.254
eGFR < 60 mL/min/1.73 m^2^	59/231	0.641	0.673	0.033 (−0.013 to 0.077)	0.365	0.379	0.183
Albuminuria ≥ 30 mg/g	80/231	0.482	0.642	0.160 (0.063–0.251)	0.333	0.503	0.230

The baseline model included age, sex, and diabetes duration. The extended model additionally included the continuous Lifestyle Compliance Index. Performance estimates were derived from stratified five-fold out-of-fold predictions. Confidence intervals for the change in ROC AUC were estimated using bootstrap resampling of paired out-of-fold predictions. Average precision should be interpreted in relation to the prevalence of each outcome. Lower Brier scores indicate improved probabilistic accuracy.

**Table 8:** Adjusted Associations Between Lifestyle Compliance and Metabolic and Renal Abnormalities.

Outcome	Participants, n	Events, n (%)	Adjusted Odds Ratio Per One-Point LCI Increase	95% CI	*p* Value
HbA1c ≥ 7%	230	118 (51.3%)	0.833	0.710–0.976	0.024
TyG index > 9.04	231	136 (58.9%)	0.757	0.645–0.889	<0.001
Obesity	231	126 (54.5%)	0.749	0.639–0.878	<0.001
eGFR < 60 mL/min/1.73 m^2^	231	59 (25.5%)	0.802	0.672–0.958	0.015
Albuminuria ≥ 30 mg/g	231	80 (34.6%)	0.716	0.606–0.845	<0.001

Each logistic regression model was adjusted for age, sex, and diabetes duration. Odds ratios below 1 indicate lower odds of the respective prevalent abnormality for each additional LCI point. The estimates represent cross-sectional associations and should not be interpreted as prospective risk reductions or causal effects. Abbreviations: aOR, adjusted odds ratio; AUC, area under the receiver operating characteristic curve; CI, confidence interval; eGFR, estimated glomerular filtration rate; HbA1c, glycated hemoglobin; LCI, Lifestyle Compliance Index; ROC, receiver operating characteristic; TyG, triglyceride-glucose.

**Figure 4:**
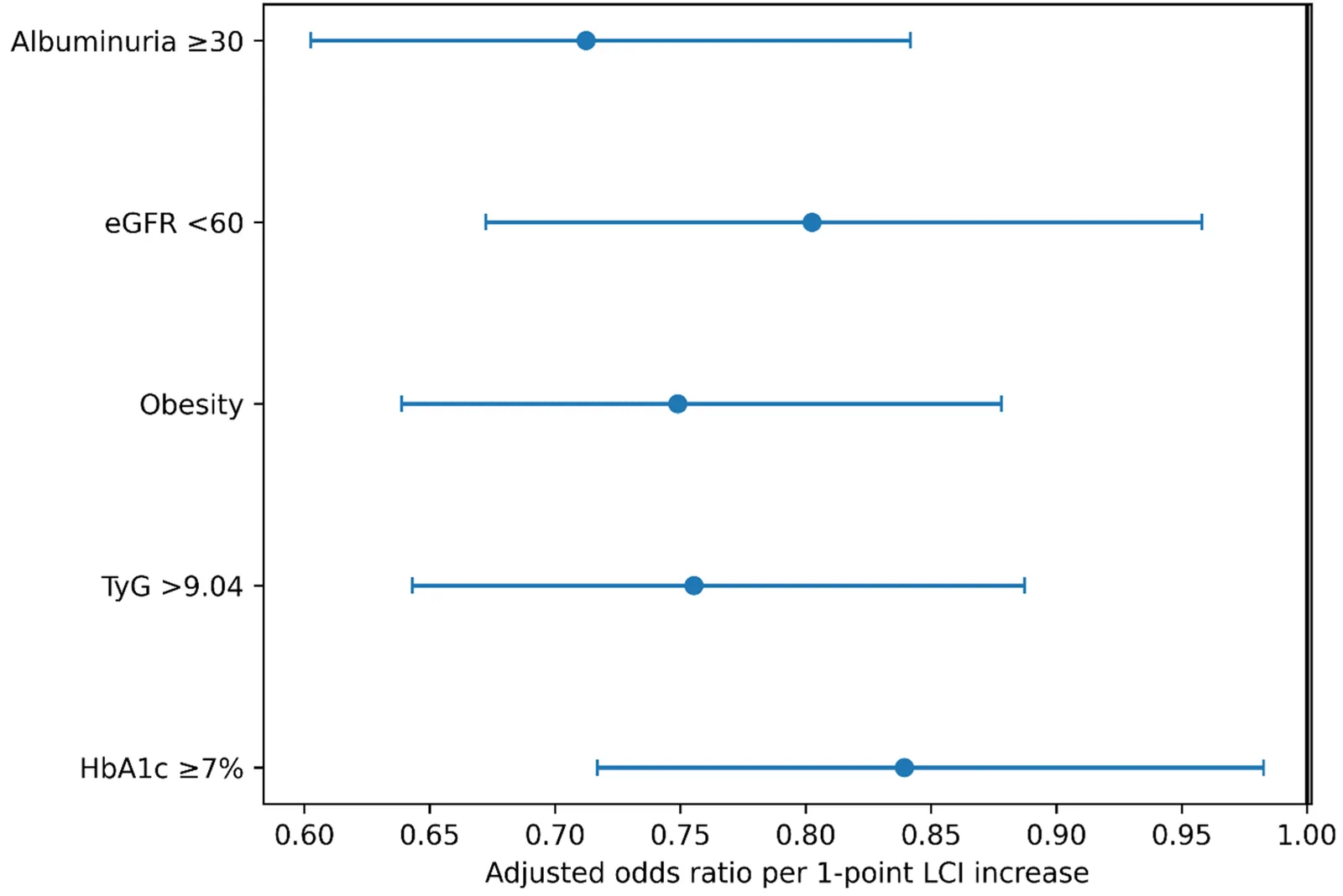
Adjusted Associations Between the Lifestyle Compliance Index and Major Metabolic and Renal Abnormalities. Forest plot showing the adjusted odds ratios (ORs) and 95% confidence intervals for the association between each one-point increase in the Lifestyle Compliance Index (LCI) and prevalent metabolic and renal abnormalities. Logistic regression models were adjusted for age, sex, and diabetes duration. Odds ratios below 1 indicate lower odds of the respective outcome with increasing lifestyle adherence. Outcomes included HbA1c ≥ 7%, TyG index > 9.04, obesity, estimated glomerular filtration rate (eGFR) < 60 mL/min/1.73 m^2^, and albuminuria ≥ 30 mg/g.

The adjusted associations between the lifestyle compliance index and major metabolic and renal abnormalities are presented in Figure 4.

## 4. Discussion

This cross-sectional study developed and evaluated an exploratory LCI comprising eight routinely assessed behaviors. Higher scores were associated with lower adiposity, more favorable triglyceride-related metabolism, better glycemic and renal measures, and lower estimated cardiovascular risk, but the index has not been validated and causal direction cannot be inferred.

### 4.1. Lifestyle Adherence Remains Suboptimal in Adults with T2DM

Despite receiving routine diabetes care, comprehensive adherence to recommended lifestyle behaviors remained relatively limited in the present sample. Although daily fruit and vegetable consumption and avoidance of sugar-sweetened beverages were common, adherence to recommendations regarding saturated fat reduction, salt restriction, and regular physical activity was substantially lower. Consequently, fewer than one-quarter of participants achieved high overall lifestyle adherence [16].

Unlike many prior tools, the LCI was designed as a formative, consultation-level summary rather than a reflective psychometric scale. The equal one-point allocation and the 0–2 physical-activity grading were pragmatic choices; they should not be interpreted as evidence that the domains have equal causal importance or that physical activity is twice as important as other behaviors.

Unlike many previous studies evaluating isolated lifestyle behaviors, the present investigation combined multiple domains into a single quantitative index. This integrated approach better reflects the multidimensional nature of modification in diabetes and may facilitate a more practical assessment of overall behavioral adherence during routine clinical practice [17].

### 4.2. Lifestyle Adherence and Metabolic Health

Participants with higher LCI scores demonstrated significantly lower body mass index, waist circumference, triglyceride concentrations, TyG index, and HbA1c values, together with higher HDL cholesterol concentrations. These associations remained significant after adjustment for age, sex, and diabetes duration, suggesting that the observed relationships were not solely explained by demographic differences [18,19].

Among all evaluated metabolic variables, the strongest association was observed between LCI and the TyG index. This finding is biologically plausible because the TyG index reflects insulin resistance and is strongly influenced by dietary habits, body weight, and physical activity [20]. Improvement in lifestyle behaviors is expected to reduce, fasting triglyceride concentrations and glucose levels, thereby improving TyG values even before substantial changes in HbA1c become apparent [21,22].

The inverse association between LCI and HbA1c was statistically significant but more modest than that observed for TyG. Glycated hemoglobin reflects glycemic exposure over approximately three months and is influenced not only by lifestyle but also by diabetes duration, pancreatic β-cell function, pharmacological treatment, and treatment adherence. Consequently, lifestyle alone cannot fully explain glycemic variability in patients with established T2DM [23,24].

The progressive reduction in obesity prevalence across increasing LCI categories further supports the internal consistency of the proposed index. Since body weight is influenced by multiple long-term behavioral factors, the observed association suggests that the LCI captures sustained lifestyle patterns rather than isolated dietary habits [25,26].

### 4.3. Renal Health and Lifestyle Compliance

The inverse association between LCI and albuminuria requires particular caution. Patients with established kidney disease may receive more restrictive dietary advice, may reduce physical activity because of frailty or comorbidity, or may otherwise change behavior after disease onset. Reverse causality and medication-related confounding therefore remain plausible.

The discrepancy between non-significant continuous UACR values and significant albuminuria prevalence may reflect skewed distributions, biological variability, influential observations, and loss of information through dichotomization. A single UACR measurement also cannot establish persistent albuminuria, which is normally confirmed with repeated testing [27,28,29].

Each additional LCI point was associated with lower adjusted odds of prevalent albuminuria, but this estimate should not be interpreted as preservation of renal function or as a prospective risk reduction.

### 4.4. Incremental Value of the Lifestyle Compliance Index

Beyond describing lifestyle behaviors, the principal objective of the study was to evaluate whether the proposed LCI provides clinically meaningful information beyond basic demographic characteristics [30].

The modest AUC gains for albuminuria, obesity, and elevated TyG index should be interpreted against weak baseline models and should not be equated with clinical usefulness. For HbA1c and renal function, pharmacological therapy, diabetes severity, and treatment adherence may explain why adding LCI produced limited incremental discrimination.

Conversely, the addition of LCI produced only modest improvements in discrimination for HbA1c ≥ 7% and reduced eGFR. Although statistically significant adjusted associations remained present, the increase in predictive performance was limited. This observation indicates that lifestyle adherence represents only one component of glycemic and renal health, both of which are also strongly influenced by diabetes duration, pharmacological therapy, genetic susceptibility, and disease severity [31].

Importantly, the LCI was never intended to replace laboratory testing or established clinical risk scores. Rather, it should be viewed as a simple behavioral assessment tool capable of identifying individuals who may benefit from more intensive lifestyle counseling and monitoring.

### 4.5. Clinical Implications

The LCI may help structure a brief behavioral conversation during outpatient care, but its clinical utility remains unproven. Because the questionnaire did not quantify hydration volume, dietary portions, food types, or objective physical activity, the score should be used only as a prompt for further assessment rather than as a treatment-triage instrument.

The findings also reinforce the concept that management of T2DM should extend beyond glycemic control alone. Current international recommendations emphasize integrated management of cardiovascular, renal, metabolic, and behavioral risk factors. The present results support this multidimensional approach by demonstrating that better lifestyle adherence is associated with improvements across several clinically relevant domains simultaneously [32].

### 4.6. Strengths and Limitations

This study used a real-world outpatient sample, standardized laboratory variables, transparent scoring rules, multiple-comparison correction, regression modelling, cross-validation, and medication-adjusted sensitivity analyses. Nevertheless, the sample was modest, the LCI categories were unvalidated, the questionnaire was self-reported, and several blood-pressure protocol details were unavailable in the retrospective database.

The LCI has no established internal consistency, test–retest reliability, criterion validity, or external validation. The index omits smoking cessation, uses data-driven cut-offs, and assigns equal weights to most domains despite their potentially different clinical importance. Treatment intensity, medication adherence, dietary composition, socioeconomic status, psychological factors, and disease severity may confound observed associations. The absence of a prospective sample-size calculation and the small high-adherence subgroup limit precision and generalizability. Selection bias is also possible because the numbers approached, eligible but not enrolled, and excluded before database entry were not available.

Future perspectives. Prospective multicenter studies should prespecify the primary outcome, recruit larger and more diverse populations, repeat lifestyle assessments longitudinally, and evaluate responsiveness to intervention. Validation should combine objective adherence measures (accelerometry or wearable activity monitoring, quantified dietary records, and measured fluid intake) with established lifestyle instruments and repeated UACR assessments. Future models should incorporate medication exposure and adherence, socioeconomic and psychosocial variables, and clinically relevant comorbidity, and should test whether LCI changes predict subsequent cardiovascular and renal outcomes.

## 5. Conclusions

In this cross-sectional sample, higher self-reported LCI scores were associated with a more favorable cardio-renal-metabolic profile. The LCI provided exploratory incremental information for obesity, elevated TyG index, and albuminuria, but the baseline albuminuria model performed below chance and the extended AUC remained modest.

The LCI should therefore be regarded as an unvalidated behavioral summary that may complement, but cannot replace, conventional clinical and biochemical assessment.

External validation against objective measures, prospective follow-up, and analyses incorporating treatment and other confounders are required before any clinical implementation is considered.

## Figures and Tables

**Table 1:** Baseline demographic, clinical, anthropometric, lifestyle, laboratory, and treatment characteristics of the study population.

Characteristic	Overall (N = 232)	Low LCI (n = 84)	Moderate LCI (n = 94)	High LCI (n = 53)	Missing, n	*p* Value
Age, years	62.58 ± 10.10	61.90 ± 10.62	63.65 ± 9.70	61.70 ± 10.06	0	0.403
Female sex, n (%)	122 (52.6%)	40 (47.6%)	48 (51.1%)	33 (62.3%)	0	0.234
Urban residence, n (%)	119 (51.3%)	40 (47.6%)	47 (50.0%)	32 (60.4%)	0	0.322
Current smoker, n (%)	29 (12.5%)	12 (14.3%)	12 (12.8%)	5 (9.4%)	0	0.704
Former smoker, n (%)	40 (17.2%)	20 (23.8%)	12 (12.8%)	7 (13.2%)	0	0.104
Diabetes duration, years	6.00 (2.00–14.00)	8.00 (1.00–14.25)	6.50 (2.00–14.75)	5.00 (2.00–11.00)	0	0.600
Hypertension, n (%)	208 (89.7%)	78 (92.9%)	84 (89.4%)	45 (84.9%)	0	0.330
Dyslipidemia, n (%)	219 (94.4%)	80 (95.2%)	88 (93.6%)	50 (94.3%)	0	0.896
Chronic kidney disease, n (%)	151 (65.1%)	60 (71.4%)	59 (62.8%)	32 (60.4%)	0	0.328
Peripheral neuropathy, n (%)	78 (33.6%)	37 (44.0%)	22 (23.4%)	19 (35.8%)	0	0.014
Diabetic retinopathy, n (%)	38 (16.4%)	20 (23.8%)	11 (11.7%)	7 (13.2%)	0	0.072
Body mass index, kg/m^2^	31.21 ± 5.70	32.98 ± 6.38	30.65 ± 5.22	29.28 ± 4.56	0	<0.001
Obesity (BMI ≥30 kg/m^2^), n (%)	127 (54.7%)	55 (65.5%)	49 (52.1%)	22 (41.5%)	0	0.019
Waist circumference, cm	106.13 ± 13.99	111.55 ± 14.43	105.05 ± 13.47	99.19 ± 10.48	0	<0.001
Systolic blood pressure, mmHg	145.87 ± 20.48	150.29 ± 20.64	144.82 ± 19.92	140.94 ± 20.35	0	0.027
Diastolic blood pressure, mmHg	86.69 ± 11.21	88.63 ± 11.71	85.28 ± 10.79	86.23 ± 11.01	0	0.130
Fasting plasma glucose, mg/dL	143.50 (118.00–188.50)	151.00 (121.75–198.50)	139.50 (119.00–177.75)	140.00 (106.00–190.00)	0	0.184
HbA1c, %	7.00 (6.25–8.60)	7.40 (6.50–8.95)	7.10 (6.30–8.57)	6.50 (6.10–7.80)	1	0.033
HbA1c ≥ 7%, n (%)	119 (51.5%)	48 (57.8%)	50 (53.2%)	20 (37.7%)	1	0.058
TyG index, index	9.15 (8.79–9.71)	9.36 (9.02–10.00)	9.12 (8.71–9.52)	8.94 (8.67–9.43)	0	<0.001
TyG index > 9.04, n (%)	137 (59.1%)	62 (73.8%)	50 (53.2%)	24 (45.3%)	0	0.001
Total cholesterol, mg/dL	183.50 (148.00–209.00)	192.50 (149.50–229.00)	174.50 (141.00–203.50)	177.00 (153.00–201.00)	0	0.231
LDL cholesterol, mg/dL	108.47 (83.36–133.81)	116.77 (81.01–144.12)	103.09 (78.06–129.92)	107.64 (87.19–126.37)	0	0.448
HDL cholesterol, mg/dL	41.00 (35.00–49.00)	39.00 (33.00–47.00)	41.00 (35.25–48.75)	45.00 (39.00–52.00)	0	0.005
Triglycerides, mg/dL	133.00 (93.00–196.50)	169.00 (110.00–259.50)	119.50 (92.00–174.25)	117.00 (83.00–153.00)	0	<0.001
Serum creatinine, mg/dL	0.83 (0.66–0.99)	0.88 (0.73–1.09)	0.85 (0.65–0.98)	0.75 (0.64–0.91)	0	0.007
eGFR, mL/min/1.73 m^2^	82.97 (59.91–94.89)	73.69 (52.56–91.60)	84.19 (61.13–94.96)	89.00 (73.46–98.71)	0	0.008
eGFR < 60 mL/min/1.73 m^2^, n (%)	59 (25.4%)	30 (35.7%)	22 (23.4%)	7 (13.2%)	0	0.011
UACR, mg/g	15.87 (11.49–46.91)	29.40 (11.91–96.23)	15.15 (11.24–43.13)	15.15 (12.66–20.41)	0	0.115
Albuminuria ≥ 30 mg/g, n (%)	80 (34.5%)	42 (50.0%)	27 (28.7%)	11 (20.8%)	0	<0.001
C-reactive protein, mg/L	3.50 (1.50–6.93)	4.25 (2.08–7.20)	3.15 (1.50–6.88)	2.90 (1.00–5.90)	0	0.115
SCORE2-Diabetes risk, %	34.87 ± 15.71	36.96 ± 16.84	35.71 ± 14.32	30.04 ± 15.64	0	0.034
Adequate hydration, n (%)	163 (70.3%)	43 (51.2%)	69 (73.4%)	50 (94.3%)	0	<0.001
Daily fruit and vegetable intake, n (%)	171 (73.7%)	40 (47.6%)	77 (81.9%)	53 (100.0%)	0	<0.001
Reduced sweets intake, n (%)	143 (61.6%)	32 (38.1%)	62 (66.0%)	48 (90.6%)	0	<0.001
Reduced saturated-fat intake, n (%)	97 (42.0%)	10 (11.9%)	45 (47.9%)	42 (79.2%)	1	<0.001
Reduced sugar-sweetened beverage intake, n (%)	184 (79.3%)	51 (60.7%)	81 (86.2%)	51 (96.2%)	0	<0.001
Alcohol abstinence, n (%)	181 (78.0%)	53 (63.1%)	77 (81.9%)	50 (94.3%)	0	<0.001
Salt intake < 5 g/day, n (%)	98 (42.2%)	13 (15.5%)	44 (46.8%)	41 (77.4%)	0	<0.001
Inactive physical activity, n (%)	134 (57.8%)	68 (81.0%)	50 (53.2%)	15 (28.3%)	0	<0.001
Health-enhancing physical activity, n (%)	31 (13.4%)	2 (2.4%)	11 (11.7%)	18 (34.0%)	0	<0.001
Any glucose-lowering medication, n (%)	213 (91.8%)	78 (92.9%)	86 (91.5%)	48 (90.6%)	0	0.886
Any antihypertensive medication, n (%)	178 (76.7%)	70 (83.3%)	69 (73.4%)	38 (71.7%)	0	0.185
Any lipid-lowering medication, n (%)	121 (52.2%)	42 (50.0%)	48 (51.1%)	30 (56.6%)	0	0.734
Insulin therapy, n (%)	75 (32.3%)	31 (36.9%)	31 (33.0%)	13 (24.5%)	0	0.318
SGLT2 inhibitor, n (%)	104 (44.8%)	38 (45.2%)	41 (43.6%)	24 (45.3%)	0	0.970
GLP-1 receptor agonist, n (%)	36 (15.5%)	12 (14.3%)	18 (19.1%)	5 (9.4%)	0	0.277

**Table 2:** Lifestyle Characteristics of the Study Population (N = 232).

Lifestyle Variable	n (%)
Adequate hydration	163 (70.3)
Daily fruit and vegetable consumption	171 (73.7)
Reduced sweets consumption	143 (61.6)
Reduced saturated fat intake	97 (42.0)
Reduced carbonated beverage consumption	184 (79.3)
Alcohol consumption	51 (22.0)
Salt intake < 5 g/day	98 (42.2)
Physical activity	
Inactive	134 (57.8)
Minimally active	67 (28.9)
Health-enhancing physical activity (HEPA)	31 (13.4)

**Table 3:** Summary Statistics of the Lifestyle Compliance Index in the Study Population.

Lifestyle Compliance Index	Value
Mean ± SD	5.03 ± 1.80
Median (IQR)	5 (4–6)
Range	1–9
Low adherence (1–4)	84 (36.4%)
Moderate adherence (5–6)	94 (40.7%)
High adherence (7–9)	53 (22.9%)

**Table 4:** Clinical and Metabolic Characteristics According to Lifestyle Compliance Index Categories.

Characteristic	Low Adherence, LCI 1–4 (n = 84)	Moderate Adherence, LCI 5–6 (n = 94)	High Adherence, LCI 7–9 (n = 53)	*p* Value
Age, years	61.90 ± 10.62	63.65 ± 9.70	61.70 ± 10.06	0.403
Diabetes duration, years	8.00 (1.00–14.25)	6.50 (2.00–14.75)	5.00 (2.00–11.00)	0.600
Body mass index, kg/m^2^	32.98 ± 6.38	30.65 ± 5.22	29.28 ± 4.56	<0.001
Obesity	55 (65.5%)	49 (52.1%)	22 (41.5%)	0.019
Waist circumference, cm	111.55 ± 14.43	105.05 ± 13.47	99.19 ± 10.48	<0.001
Systolic blood pressure, mmHg	150.29 ± 20.64	144.82 ± 19.92	140.94 ± 20.35	0.027
Diastolic blood pressure, mmHg	88.63 ± 11.71	85.28 ± 10.79	86.23 ± 11.01	0.130
Fasting plasma glucose, mg/dL	151.00 (121.75–198.50)	139.50 (119.00–177.75)	140.00 (106.00–190.00)	0.184
HbA1c, %	7.45 (6.50–9.10)	7.10 (6.30–8.57)	6.50 (6.10–7.80)	0.025
HbA1c ≥ 7%	48 (57.1%)	50 (53.2%)	20 (37.7%)	0.075
TyG index	9.36 (9.02–10.00)	9.12 (8.71–9.52)	8.94 (8.67–9.43)	<0.001
TyG index > 9.04	62 (73.8%)	50 (53.2%)	24 (45.3%)	0.001
Total cholesterol, mg/dL	192.50 (149.50–229.00)	174.50 (141.00–203.50)	177.00 (153.00–201.00)	0.231
LDL cholesterol, mg/dL	116.77 (81.01–144.12)	103.08 (78.06–129.92)	107.64 (87.19–126.37)	0.448
HDL cholesterol, mg/dL	39.00 (33.00–47.00)	41.00 (35.25–48.75)	45.00 (39.00–52.00)	0.005
Triglycerides, mg/dL	169.00 (110.00–259.50)	119.50 (92.00–174.25)	117.00 (83.00–153.00)	<0.001
Serum creatinine, mg/dL	0.88 (0.73–1.09)	0.85 (0.65–0.98)	0.75 (0.64–0.91)	0.007
eGFR, mL/min/1.73 m^2^	73.69 (52.56–91.60)	84.20 (61.14–94.96)	89.00 (73.46–98.71)	0.008
eGFR < 60 mL/min/1.73 m^2^	30 (35.7%)	22 (23.4%)	7 (13.2%)	0.011
UACR, mg/g	29.40 (11.91–96.23)	15.15 (11.24–43.13)	15.15 (12.66–20.41)	0.115
Albuminuria ≥ 30 mg/g	42 (50.0%)	27 (28.7%)	11 (20.8%)	<0.001
C-reactive protein, mg/L	4.25 (2.08–7.20)	3.15 (1.50–6.88)	2.90 (1.00–5.90)	0.115
SCORE2-Diabetes, %	36.96 ± 16.84	35.71 ± 14.32	30.04 ± 15.64	0.034

Data are presented as mean ± standard deviation, median (interquartile range), or number (percentage), as appropriate. One-way analysis of variance was used for approximately normally distributed continuous variables, the Kruskal–Wallis test for non-normally distributed variables, and Pearson’s chi-square test or Fisher’s exact test for categorical variables, as appropriate. Significant pairwise comparisons were examined using Tukey’s test after ANOVA or Mann–Whitney U tests with Holm correction after the Kruskal–Wallis test. HbA1c was available for 230 participants in this table after exclusion of one invalid LCI response and one clinically implausible HbA1c value.

## Data Availability

The original contributions presented in this study are included in this article. Further inquiries can be directed to the first authors.

## Abbreviations

The following abbreviations are used in this manuscript:

Abbreviation	Definition
AUC	Area under the curve
BMI	Body mass index
CI	Confidence interval
CRP	C-reactive protein
DPP-4	Dipeptidyl peptidase-4
eGFR	Estimated glomerular filtration rate
FIB-4	Fibrosis-4 index
GLP-1	Glucagon-like peptide-1
HbA1c	Glycated hemoglobin
HDL	High-density lipoprotein
IQR	Interquartile range
LDL	Low-density lipoprotein
OR	Odds ratio
ROC	Receiver operating characteristic
SD	Standard deviation
SGLT2	Sodium–glucose cotransporter-2
T0	Baseline assessment
T1	Six-month follow-up assessment
T2DM	Type 2 diabetes mellitus
TyG	Triglyceride–glucose index
UACR	Urinary albumin-to-creatinine ratio
